# Investigation of Correlated Internet and Smartphone Addiction in Adolescents: Copula Regression Analysis

**DOI:** 10.3390/ijerph17165806

**Published:** 2020-08-11

**Authors:** Minji Lee, Sun Ju Chung, Youngjo Lee, Sera Park, Jun-Gun Kwon, Dai Jin Kim, Donghwan Lee, Jung-Seok Choi

**Affiliations:** 1 Department of Statistics, Ewha Womans University, Seoul 03760, Korea; hsjiho2104@gmail.com; 2 Department of Psychiatry, SMG-SNU Boramae Medical Center, Seoul 07061, Korea; sunjujung1991@gmail.com; 3 Department of Statistics, Seoul National University, Seoul 08826, Korea; youngjo@snu.ac.kr; 4 I Will Center, Seoul Metropolitan Boramae Youth Center, Seoul 07062, Korea; seramanim@boramyc.or.kr (S.P.); jun@boramyc.or.kr (J.-G.K.); 5 Department of Psychiatry, Seoul St. Mary’s Hospital, College of Medicine, The Catholic University of Korea, Seoul 06591, Korea; kdj922@catholic.ac.kr; 6 Department of Psychiatry and Behavioral Science, Seoul National University College of Medicine, Seoul 03080, Korea

**Keywords:** internet addiction, smartphone addiction, copula regression

## Abstract

Internet and smartphone addiction have become important social issues. Various studies have demonstrated their association with clinical and psychological factors, including depression, anxiety, aggression, anger expression, and behavioral inhibition, and behavioral activation systems. However, these two addictions are also highly correlated with each other, so the consideration of the relationship between internet and smartphone addiction can enhance the analysis. In this study, we considered the copula regression model to regress the bivariate addictions on clinical and psychological factors. Real data analysis with 555 students (age range: 14–15 years; males, *N* = 295; females, *N* = 265) from South Korean public middle schools is illustrated. By fitting the copula regression model, we investigated the dependency between internet and smartphone addiction and determined the risk factors associated with the two addictions. Furthermore, by comparing the model fits of the copula model with linear regression and generalized linear models, the best copula model was proposed in terms of goodness of fit. Our findings revealed that internet and smartphone addiction are not separate problems, and that associations between them should be considered. Psychological factors, such as anxiety, the behavioral inhibition system, and aggression were also significantly associated with both addictions, while ADHD symptoms were related to internet addiction only. We emphasize the need to establish policies on the prevention, management, and education of addiction.

## 1. Introduction

The spread of the Internet and smartphones has the advantage of rapidly offering information and improving living conditions. Moreover, Korea has seen a steady rise in the Internet penetration rate since the launch of internet commercial services in 1994, along with the growth rate of smartphones among teenagers [1,2]. Because adolescents generally have less self-control and spend more time using smartphones compared to adults, the probability of potential addiction is observed as higher in adolescents than adults. [3]. Recent work has demonstrated that the excessive use of online gaming is linked to poorer social skills and an absence of real-life relationships [4], while the addictive use of social media leads to problems strongly associated with symptoms of attention-deficit/hyperactivity disorder (ADHD) [5]. Furthermore, low levels of self-control and emotional control issues have been associated with adolescent internet addiction [6]. An over-reliance on smartphones can even result in psychological problems, such as anxiety and depression [7]. In addition to this, physical problems have also been linked to long-term smartphone usage, including muscle ache and headaches, which subsequently deteriorate sleep quality [8].

The main function of smartphones is the operation of internet-based applications. Smartphones are known as the “handheld internet” because they provide personalized services anywhere in real time, unlike generally fixed desktops [9]. Thus, the Internet and smartphones are used in sync, and although internet and smartphone addictions have unique characteristics, such as that males are at more risk of internet addiction, while females are more prone to smartphone addiction [9], they also share behavioral addiction features based on information technology (IT) [10]. It is well-known that several clinical characteristics, such as depression, anxiety, aggression, and impulsivity have a positive relationship with both addictions [11,12,13,14]. Using these shared or similar properties, smartphone addiction criteria were developed by referring to internet addiction criteria [15]. A study in Japan also determined that smartphone and internet addictions were significantly associated [16]. Previous studies have investigated the positive association between internet and smartphone addictions. For example, smartphone addiction risk groups were observed to use mobile messenger, social networking services, and the Internet for significantly longer periods of time compared to non-risk groups [17]. The smartphone addition scale (SAS) was found to be a significant factor of internet addition using stepwise multiple regression. Conversely, when SAS was used as a response variable, internet addiction was exhibited as a relevant factor [10].

However, up until now, the majority of studies have used multiple or binary logistic regression in order to analyze internet and smartphone addiction. Such analysis methods assume that internet and smartphone addiction are independently observed [10,17]. The copula regression model can be easily applied when the response variables have different distribution patterns and are correlated in multivariate data [18]. In this paper, we applied copula regression analysis for the prediction of internet and smartphone addiction simultaneously. More specifically, we used data collected from Korean adolescents on internet and smartphone addictions and compared our proposed model with regression and generalized linear models. In addition, we explained the consequences of the model selection criteria. Finally, we determined the optimum model and significant features associated with internet and smartphone addiction, such as depression, anxiety, aggression, anger expression, the behavioral inhibition system, and behavioral activation system. We chose these variables since these are known as the most representative psychological characteristics associated with internet and smartphone addiction, which lots of previous research have revealed.

## 2. Materials and Methods

### 2.1. Participants

This study was approved by the Institutional Review Board of Seoul St. Mary’s Hospital, Seoul, Republic of Korea (KC13ONSI0080, 8 April 2013), and all subjects provided written informed consent prior to participation. Data was collected from 714 (males, *N* = 389; females, *N* = 325) middle school students (age range: 14–15 years) in Seoul, South Korea. All participants received an explanation about the research and completed self-administered questionnaires. Participants were offered gift certificates as a reward for their participation. The survey included basic information, such as the age, sex, and drinking and smoking characteristics of participants, and medical records of psychiatric hospitals were checked. Out of the 714 observations, we eliminated 159 samples due to missing values for the Internet Addiction Test (IAT) and Smartphone Addiction Scale (SAS). The data for the final sample used for analysis included 295 males and 260 females, with total of 555 subjects.

### 2.2. Measures

#### 2.2.1. Young’s Internet Addiction Test (Y-IAT)

The Y-IAT, developed by Kimberly Young, is one of the most utilized diagnostic instruments for Internet addiction. It is based on a five-point scale (from “1 = very rarely” to “5 = very frequently”). The Y-IAT has 20 items, including questions such as, “How often do you find that you stay online longer than you intended?” and “How often do you neglect household chores to spend more time online?”. It contains six subscales: cognitive conspicuousness (Y1), overuse (Y2), neglect of duty (Y3), expectation (Y4), lack of control (Y5), and neglect of social activities (Y6). A Cronbach’s alpha coefficient for Y-IAT of 0.939 has previously been reported. Based on previous research, the total scores were calculated according to Young’s procedure, with possible scores for all 20 items ranging from 20 to 100. A total score of 20–39 represents average internet usage with self-control. A score of 40–69 denotes excessive internet usage with experience of frequent problems, and 70–100 represents serious problems due to internet usage [19].

#### 2.2.2. Smartphone Addiction Scale (SAS)

The SAS was used to assess the degree of problematic smartphone usage. The SAS includes 33 items, such as questions of “feeling calm or cozy while using a smartphone” and “missing planned work due to smartphone use” with a six-point response format, ranging from “1 = strongly disagree” to “6 = strongly agree”. The SAS is composed of and calculated from six subscales: daily life disturbance (S1), positive anticipation (S2), withdrawal (S3), cyberspace-oriented relationship (S4), overuse (S5), and tolerance (S6). The SAS has previously demonstrated an internal consistency with a Cronbach’s alpha coefficient of 0.967. The higher the total SAS score, the higher the degree of addictive behavior related to smartphones [20].

#### 2.2.3. Psychosocial Measures

The Beck Depression Inventory (BDI) is a self-rated measure that consists of 21 questions. It is used to quantify the severity of a particular symptom experienced during the past week and includes cognitive, emotional, motivational, and physical features of depression. A total score of 0–13 is considered to denote minimal depression, 14–19 mild depression, 20–28 moderate depression, and 29–63 severe depression. The Korean version has previously been validated with a Cronbach’s alpha coefficient of 0.85 [21,22].

The Beck Anxiety Inventory (BAI) is another self-rated measure used to rate the seriousness of anxiety and consists of questions on, for example, sensitivity and physical sense. In total, 21 questions and a four-point scale (0 = ‘‘not at all’’ to 3 = ‘‘it bothered me severely’’) are used. Scores for the 21 items are summed up to calculate a single anxiety score. Previous research has demonstrated internal consistency with a Cronbach’s alpha coefficient of 0.93 [23].

The Behavioral Inhibition System (BIS) and Behavioral Activation System (BAS) scales were designed based on Gray’s theory of biomechanical nature, whereby the pursuit of avoidance of a punishment system, and sensitivity and approach to a compensation system are evaluated. This scale consists of 20 questions rated on a four-point scale from “totally agree” to “totally disagree”. In particular, the BIS scale contains seven items on anticipated punishment, and the BAS scale has 13 items [24].

The short form of the Conners-Wells’ Adolescent Self-Report Scale (CASS) evaluates ADHD symptoms, concentrating on cognitive- and hyperactivity-related issues. It consists of 27 items that rate problems of ADHD symptoms based on a four-point scale, ranging from 0 (not true) to 3 (very often). This measure has demonstrated internal consistency with a Cronbach’s alpha coefficient of 0.88 [25].

The Aggression Questionnaire (AQ) consists of 29 questions on a five-point Likert scale from 1 (uncharacteristic of me) to 5 (very characteristic of me) as a measure of aggressive behavior. The questions are divided into four domains: physical or verbal aggression, anger, and hostility. This measure has demonstrated internal consistency with a Cronbach’s alpha coefficient of 0.889 [26].

The State–Trait Anger Expression Inventory (STAXIE) developed by Spielberger [27] was designed to measure how often the respondent experiences anger on the same scale. It consists of 20 questions, and each item is evaluated with a four-point scale [27].

#### 2.2.4. Internet/Smartphone Usage Pattern Survey

We surveyed internet and smartphone usage patterns on weekdays and weekends. A recent review found that problematic internet use is most common in adolescents, as they are more vulnerable to internet addiction due to their limited ability to control their enthusiasm for internet activities [10]. Moreover, first using a smartphone at a younger age was found to be significantly associated with several smartphone addiction symptoms [28]. Following this, the initial use of the internet and smartphones was added to our survey. In addition, participants answered questions related to gaming patterns based on a study on internet-based games that determined game play as an important indicator of the potential risk of internet gaming addiction [28].

### 2.3. Statistical Analysis

In order to model Y-IAT and SAS simultaneously, we used the copula regression model. Copula models are useful for describing response variables that are not normally distributed and that have the dependence structure. In addition, different marginal distributions for response variables are allowed.

Let $F_{Y_{1}}\left(y_{1}\right)\ldots F_{Y_{d}}\left(y_{d}\right)$ denote the marginal distribution of the multivariate responses $Y_{1},\ldots ,\;Y_{d}$. From Sklar’s Theorem [29], their joint distribution function can be created by referring to a unique copula function  C, as

(1) $$F_{Y_{1},\ldots ,Y_{d}}\left(y_{1},\ldots ,y_{d}\right)=C\left[F_{Y_{1}}\left(y_{1}\right),\ldots ,F_{Y_{d}}\left(y_{d}\right)\right].$$

If the marginal distributions are continuous, then the copula function C is unique. In this paper, we set *d* = 2 because the outcome variables (Y-IAT and SAS) are two-dimensional. The Gaussian copula is a model generated from the multivariate normal distribution using the inverse normal transformation,

$$C\left(u_{1},u_{2};\rho \right)=\Phi_{2}\left(\Phi^{-1}\left(u_{1}\right),\Phi^{-1}\left(u_{2}\right);\rho \right),$$

where Φ() is the standard normal cumulative distribution and $\Phi_{2}$() is the bivariate normal cumulative distribution with 0 mean, unit variances, and correlation parameter ρ [30]. Similarly, the Student-t copula [31], with the degrees of freedom parameter ν >2, is derived with bivariate t distribution, $t_{\Sigma ,\;v}\left(\right)$ with the density

$$f\left(y\right)=\frac{\Gamma \left(\frac{\nu +2}{2}\right)}{\Gamma \left(\frac{\nu }{2}\right)\sqrt{{\left(\pi \nu \right)}^{2}\left|\Sigma \right|}}{\left(1+\frac{y^{T}\Sigma^{-1}y\;}{\nu }\right)}^{-\frac{\nu +2}{2}},$$

where $y={\left(y_{1},y_{2}\right)}^{T}$.

For applying the copula function in the regression context, each marginal distribution $F_{Y_{1}}\left(y_{1}\right)$ = $F_{1}\left(y_{i1};X_{i1,}\beta_{1}\right)$ and $F_{Y_{2}}\left(y_{2}\right)$ = $F_{2}\left(y_{i2};X_{i2,}\beta_{2}\right)$ can be modeled with the explanatory variables $X_{i1}$ and $X_{i2}\;$ and the regression coefficients $\beta_{1}$ and $\beta_{2}$. Therefore, in order to estimate unknown parameters, we can maximize the likelihood constructed by the joint distribution

$$F_{Y_{1},Y_{2}}\left(y_{i1},\;y_{i2};X_{i1},\;X_{i2},\beta_{1},\beta_{2},\;\theta \right)=C\left[F_{1}\left(y_{i1};X_{i1,}\beta_{1}\right),F_{2}\left(y_{i2};X_{i2,}\beta_{2}\right);\theta \right],$$

where the parameter θ captures the degrees of association between two marginals. In summary, following [18], fitted outcome variables can be derived based on the copula regression model with the following steps: (a) Determine the model for the joint distribution of the outcome variables and covariates; (b) estimate the distribution and parameters of the model (marginal and copula); and (c) estimate the conditional mean of the outcome variables given the covariates. We compared our proposed model with a linear regression model [32] and a generalized linear model (GLM) [33] for the outcome variables of Y-IAT and SAS, and covariates of psychosocial measures and Internet/smartphone usage patterns.

## 3. Results

**Figure 1:**
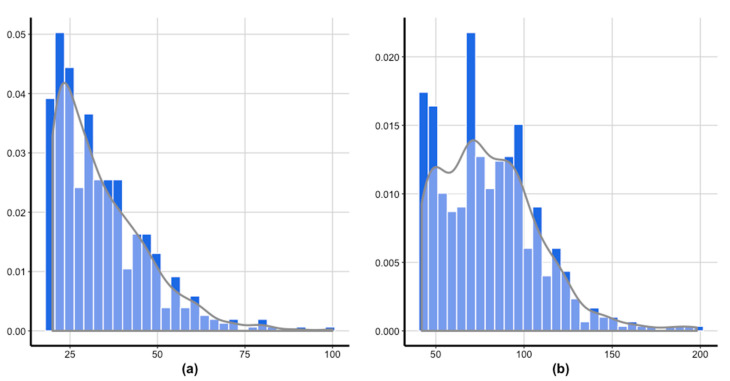
Histogram and density plot of the response variables: (**a**) Y-IAT; (**b**) SAS.

**Figure 2:**
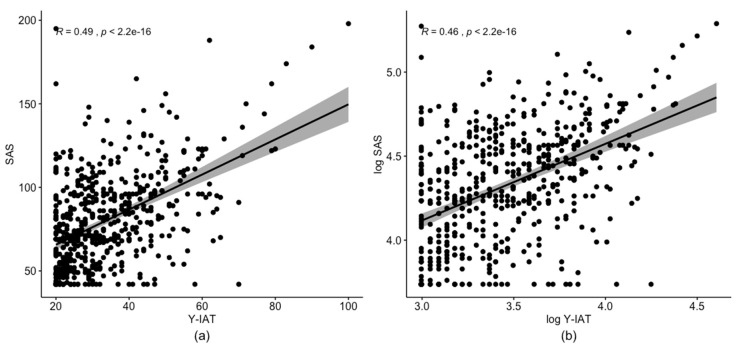
Scatter plot and correlation (**a**) between Y-IAT and SAS; (**b**) between log-transformed Y-IAT and log-transformed SAS.

First, we investigated the characteristics of the outcome variables (Y-IAT and SAS) and psychological measures. Table 1 shows the mean, standard deviations of the measures, and their correlations. Figure 1 demonstrates that Y-IAT and SAS are non-negative and skewed to the right. Figure 2 shows that there is a strong and positive correlation between IAT and SAS. In particular, the Pearson correlation between the two variables is $\rho_{y_{1},y_{2}}=0.49$ (*p*-value < 0.001). Moreover, a strong association is still observed following the log-transformation of IAT and SAS. Thus, in order to jointly model IAT and SAS, the dependence structure between IAT and SAS must be considered.

Figure 1 and Figure 2 suggest that right-skewed distributions, such as the gamma, log-normal, and Weibull distribution are potential candidates for marginal distributions. Table 2 and Table 3 report the estimated parameters of the fitted distributions of Y-IAT and SAS, respectively, using R-package *fitdistrplus* [34]. The standard error, Akaike information criterion (AIC), and Bayesian information criterion (BIC) for the log-normal (AIC of IAT = 4023.95, AIC of SAS = 4917.60) and gamma (AIC of IAT = 4063.73, AIC of SAS = 4922.90) distributions were smaller than that of the Weibull distribution (AIC of IAT = 4168.62, AIC of SAS = 4978.22). Therefore, herein, we assume the distributions of IAT and SAS to be log-normal and gamma, respectively.

Prior to conducting the copula regression model, we first determined an appropriate copula distribution without explanatory variables using the R (version 3.5.1) package *VineCopula*. This package is able to select a suitable copula family for given dependence data, and the relevant parameter estimates are calculated by maximum likelihood estimation. Moreover, the optimal distribution is selected based on the AIC and BIC [35]. Based on this, we determined the optimal copula distribution to be a t-distribution with three degrees of freedom. Goodness-of-fit tests for the selected copula function were performed using *gofCopula* [36].

For each model, the AIC was calculated for variable selection by backward elimination [37]. As the linear regression and generalized linear models assume the IAT and SAS to be independent, the total AIC for each variable is calculated as the sum of the AIC values in two models. Table 4 reports the AIC of the copula regression model with varying marginal and copula values. In terms of AIC, the copula regressions performed better than the linear regression and generalized linear models. For the copula models, the optimum scenario was for the IAT with a log-normal distribution, SAS with a gamma distribution, and the copula with a t-distribution.

**Figure 3:**
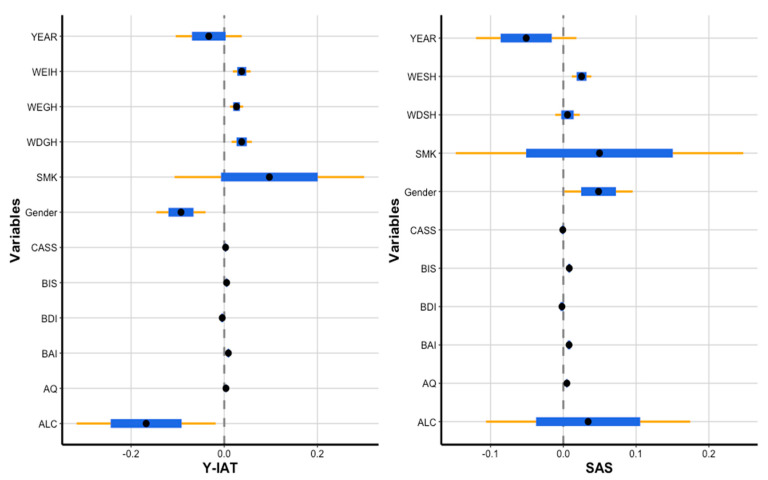
The plots of regression coefficients when fitting the t-copula, (**a**) Internet Addiction Test (Y-IAT) part and (**b**) Smartphone Addiction Scale (SAS) part.

Table 5 and Figure 3 present the fitted results of the best model, the t-copula regression model. The response variables IAT and SAS were dependent on each other. For the IAT, the gender coefficient was a negative value (reference variable = male). However, the SAS female regression coefficient exhibited a high, significantly positive value (*p* < 0.05). Among the psychological factors, BAI, BIS, and AQ were significantly associated with both addictions, while CASS affected to IAT only. As well as the psychological measures, internet and smartphone usage patterns from the survey were also significant.

## 4. Discussion

We explored regression models for internet and smartphone addiction, with the relevant predictors. In order to account for the dependence between the two addictions, we used the copula regression method and demonstrated that this model was better than conventional methods in terms of AIC. Most studies generally predict addiction using multiple linear or logistic regressions, without considering the association between variables [10,17]. In addition, the marginal correlation between internet and smartphone addiction has been tested in previous studies [16]. Similar to previous results, we found that, in the final model for internet addiction, the gaming hour variables during weekdays and weekend were significant. Moreover, psychological variables, such as anxiety, behavioral inhibition, and aggression are potential risk factors of both addictions. Several studies have reported anxiety and aggression as significant risk factors of the smartphone [7,38] and internet [11,39] addictions. BIS sensitivity is also a known key influence on internet addiction [40,41,42], and individuals with a smartphone addiction have demonstrated higher BIS values compared to non-addicted users [43]. Thus, our findings act as additional evidence that these factors, which may increase vulnerability to internet and smartphone addictions, independently increase the risk of both addictions. Our results indicate that adaptive coping strategies must be developed, specifically including the mood regulation of individuals with high levels of anxiety, aggression, and BIS reactivity, in order to prevent internet and smartphone addiction.

Additionally, the present study identified the fact that internet addiction encompasses broader factors related to both addictions, in that there are more independent factors which predict internet addiction rather than smartphone addiction apart from common predictors of both addictions. It was revealed that symptoms of depression and ADHD were significantly associated with internet addiction, although the powers of significance were low, whereas no significant relationship was observed for smartphone addiction in this study. Similar to previous research [44,45], we found that ADHD symptoms positively predicted internet addiction. This finding demonstrates the necessity to assess and treat comorbidities associated with internet addiction. Furthermore, in a recent study using structural equation modeling [46], affective components, such as depression and anxiety, were significantly associated with both internet addiction and smartphone addiction, whereas aggression, the expression of anger, and ADHD symptoms affected only internet addiction. Adolescents are more easily addicted to the Internet, or playing internet games to gain rewards instantly and express their negative feelings through externalizing problems, while adolescents with depression or anxiety might use the Internet or smartphone to avoid or reduce their negative mood [46]. Therefore, internet addiction might be related with more psychological and clinical factors rather than smartphone addiction. However, in the present study, depression was negatively associated with internet addiction, inconsistent with previous results [47,48,49]. Because the correlation between anxiety and depression was positively high in this study, and the symptom of anxiety is very significantly related to addictions with a positive sign, there may have been a confounding effect due to the multicollinearity. It is necessary to confirm relationships between depression and internet addiction in the further study with a larger sample.

Our findings regarding gender are in line with previous studies which indicate the different patterns of internet and smartphone usage by gender [50,51]. Lee et al. (2018) [50] explored internet and smartphone usage patterns among adolescents, and revealed that female students exhibited problematic usage trends in smartphones, but lower levels of severity for internet usage compared to male students [50]. These findings suggest that gender is a significant predictor of internet and smartphone addictions, and particularly indicate females as more susceptible to smartphone addiction than internet addiction. Such gender-specific results may be explained by previous research. Studies have revealed that females tend to use mobile phones and the Internet for social communication, while males use the Internet for leisure activities and interests, such as gaming and entertainments [52,53]. Thus, gender differences must be considered for addiction interventions, with particular focus on gender-specific characteristics for internet and smartphone addictions.

The results of our study also demonstrated that the time spent using the Internet on weekdays, and gaming hours during both weekdays and the weekend were significant predictors of internet addiction. Similarly, the time spent using smartphones on the weekend positively predicted smartphone addiction. That is, the more time spent on each media type, the higher the severity of the addiction. Consequently, these results suggest that the quantitative index of each media type reflects the level of addiction. In particular, the fact that online gaming generally accounts for a great portion of internet usage [12,54] offers an explanation of the results, and implies that online gaming usage must be dealt with within internet addiction treatments. Our study is limited by our data. For example, our dataset was gathered from an adolescent age group. Thus, difficulties can arise in generalizing our results because adults’ internet and smartphone addiction may have different patterns due to other social problems, such as gambling. Hence, we propose further studies that can extend the scope of the survey, with greater gender and age ranges in order to allow for generalizations of internet and smartphone usage patterns. Furthermore, these real examples have missing values of addiction scores. In this paper, we simply used completed-case analysis because we found no meaningful difference between the distributions of psychological variables of samples with or without addiction values. If the non-response patterns are not ignorable, more care is necessary.

## 5. Conclusions

In this paper, we considered a regression model where the marginal distributions of each response variable are different and are correlated with each other. We selected and predicted the best model considering the correlation between internet and smartphone addiction. Consequently, we found that the copula regression provides a better fit compared to the linear regression and generalized linear models. Our findings reveal that internet and smartphone addiction are not separate problems; the association between IAT and SAS should be considered. Furthermore, we found the potential risk factors associated with the two addictions, so we emphasized the need to establish policies on the prevention, management, and education of addiction. Though current interventions of both addictions usually take place independently, this suggests there is a necessity for interventions on both addictions in future, even if there is only one risk of either, keeping in mind the possibility of it developing into another one.

## Figures and Tables

**Table 1:** Descriptive statistics of addiction and psychosocial measures.

Variable	Mean	SD	Correlations							
			Y_IAT	SAS	BDI	BAI	BIS	CASS	AQ	STAXI_E
Y_IAT	33.88	13.05	1	0.49	0.23	0.44	0.32	0.42	0.4	0.23
SAS	80.25	27.94		1	0.39	0.48	0.39	0.39	0.49	0.26
BDI	7.46	7.82			1	0.65	0.31	0.52	0.5	0.33
BAI	6.46	8.56				1	0.3	0.55	0.52	0.3
BIS	63.55	8.34					1	0.44	0.33	0.14
CASS	17.91	12.59						1	0.6	0.44
AQ	63.42	20.06							1	0.53
STAXI_E	53.28	11.39								1

**Table 2:** Parameter estimates of probability distributions of Y-IAT.

Parameters		Estimate	SE	AIC	BIC
**Gamma**	**shape**	8.073	0.488	4063.73	4072.26
	**rate**	0.237	0.015		
**Log-normal**	**mean**	3.464	0.015	4023.95	4032.48
	**standard deviation**	0.346	0.011		
**Weibull**	**shape**	2.700	0.083	4168.22	4177.15
	**scale**	38.262	0.658		

Abbreviations: SE, Standard error; AIC, Akaike information criterion; BIC, Bayesian information criterion; Y-IAT, Young’s Internet Addiction Test.

**Table 3:** Parameter estimates of probability distributions of SAS.

Parameters		Estimate	SE	AIC	BIC
**Gamma**	**shape**	8.073	0.488	4063.732	4072.262
	**rate**	0.237	0.015		
**Log-normal**	**mean**	3.464	0.015	4023.95	4032.481
	**sd**	0.346	0.011		
**Weibull**	**shape**	2.70	0.083	4168.623	4177.154
	**scale**	38.262	0.658		

Abbreviations: SE, Standard error; AIC, Akaike information criterion; BIC, Bayesian information criterion, SAS, Smartphone Addiction Scale.

**Table 4:** Model comparison results.

Linear Regression			GLM			Copula			
IAT	SAS	AIC	IAT	SAS	AIC	IAT	SAS	Copula	AIC
Norm.	Norm.	8605.3	Log.	Log.	8587.1	Log.	Gam.	Norm.	8363.1
Log.	Log.	8421.8	Gam.	Gam.	8453.2	Log.	Log.	t	8362.2
Norm.	Log.	8572.6	Log.	Gam.	8567.4	Log.	Gam.	t	8348.5
Log.	Norm.	8454.5	Gam.	Log.	8472.9	Gam.	Log.	t	8373.4

Abbreviations: Norm., Normal distribution; Log., Log normal distribution; Gam., Gamma distribution; GLM, generalized linear model.

**Table 5:** Parameter estimates of the t-copula regression model.

IAT	Estimate	SE	*p*-Value	SAS	Estimate	SE	*p*-Value
**Intercept**	69.1356	72.4595	0.34	**Intercept**	114	71.86	0.11267
**GENDER**	−0.0929	0.027	0.0006	**GENDER**	0.0484	0.0239	0.0428
**BDI**	−0.0044	0.0021	0.0363	**BDI**	−0.0019	0.0021	0.3732
**BAI**	0.0089	0.0019	0.0000	**BAI**	0.0079	0.0019	0.0000
**BIS**	0.0052	0.0016	0.0009	**BIS**	0.0082	0.0015	0.0000
**CASS**	0.0029	0.0013	0.0307	**CASS**	−0.0007	0.0013	0.6154
**AQ**	0.0037	0.0008	0.0000	**AQ**	0.0049	0.0008	0.0000
**WDGH**	0.0375	0.0111	0.0008	**WDSH**	0.0057	0.0086	0.5077
**WEGH**	0.0264	0.0073	0.0003	**WESH**	0.0251	0.0069	0.0003
**WDIH**	0.0376	0.0098	0.0001	**YEAR**	−0.051	0.0352	0.1478
**YEAR**	−0.0332	0.0362	0.3595	**ALC**	0.0341	0.0716	0.6343
**ALC**	−0.1677	0.0762	0.0277	**SMK**	0.0497	0.1008	0.622
**SMK**	0.0969	0.1039	0.3508				
AIC	8348.471						
Corr.	0.364 (0.288, 0.429)						

Abbreviations: SE, standard error; Corr., Correlation; AIC, Akaike information criterion; WDGH, Weekday daily gaming hours; WEGH, Weekend daily gaming hours; WDIH, Weekday daily Internet usage hours; WDSH, Weekday daily Smartphone usage hours; WESH, Weekend daily Smartphone usage hours; YEAR, Birth year, ALC, Alcohol Drinking, SMK, Smoking.
